# The role of five big personality traits and entrepreneurial mindset on entrepreneurial intentions among university students in Saudi Arabia

**DOI:** 10.3389/fpsyg.2022.964875

**Published:** 2022-10-19

**Authors:** Basheer M. Al-Ghazali, Syed Haider Ali Shah, M. Sadiq Sohail

**Affiliations:** ^1^Interdisciplinary Research Center for Finance and Digital Economy (IRC-FDE), Dammam Community College, King Fahd University of Petroleum and Minerals, Dhahran, Saudi Arabia; ^2^Business Studies Department, Bahria University Islamabad, Islamabad, Pakistan; ^3^Department of Management and Marketing, Interdisciplinary Research Center for Finance and Digital Economy (IRC-FDE), KFUPM Business School, King Fahd University of Petroleum and Minerals, Dhahran, Saudi Arabia

**Keywords:** five big personality traits, entrepreneurial mindset, entrepreneurial passion, entrepreneurial self-efficacy, entrepreneurial intention

## Abstract

The big five personality traits and entrepreneurial mindset (EM) are crucial individual-level elements that determine entrepreneurial intention (EI). This study examines the impact of big five personality traits and EM, on EI using the theory of planned behavior. Besides, this study examined the role of entrepreneurial self-efficacy (ESE) and attitude toward entrepreneurship (ATE) influences EI. To achieve the research objectives, a quantitative approach was used. Structural equation modeling (SEM) and path analysis were conducted using SmartPLS software. Data were collected from 270 respondents through online questionnaires. Findings of the study revealed that big five personality traits influence ESE and ATE which led to EI. Finally, the moderating role of entrepreneurial passion was also found to have strong effect on influence ESE and ATE. This study offers evidence and insights that academics, educators, and others involved in the creation or expansion of entrepreneurial knowledge can use as a reference point.

## Introduction

Entrepreneurship plays a critical part in a country’s economic growth and development (Katz, 2003; Davey et al., 2016), Individuals can use it as a feasible career option. It provides unemployed youth with a key path to self-sufficiency by allowing them to start their own business (Bell and Bell, 2016). In governments failing to create jobs in such critical times, it is critical to create new jobs for young people as a self-employment (Kuckertz and Wagner, 2010). The act of starting a business is preceded by “EIs” as an individual involved in taking advantage of opportunities that are available (Liao et al., 2022). The literature on entrepreneurship has emphasized the importance of intentions in deciding whether or not to start a new business (Bowen and Hisrich, 1986; Hisrich, 1992; Krueger et al., 2000; Kim and Aldrich, 2005; Bögenhold and Fachinger, 2010, 2014; Dieter and Uwe, 2011; Aldrich, 2012; Kautonen et al., 2015; Hisrich and Ramadani, 2017; Cui, 2021). The desire to pursue a career as an entrepreneur is seen to be a key factor in determining the success of new companies. However, very few studies have considered the factors that influence individual intentions in context of Saudi Arabia. To gain greater knowledge of the factors that impact entrepreneurial intent could help ventures evolve more successfully, especially for university students, who are more likely to pursue self-employment that has a large impact on economic growth than those without a university education (Robinson and Sexton, 1994). According to the literature, researchers advocated that there is a link between entrepreneurial intent and personality traits (Almeida et al., 2014). Furthermore, personality traits are becoming a more prominent research focus in the entrepreneurial and psychology literature. However, there is mixed opinion on the significance of personality in predicting entrepreneurial intent (Baron and Shane, 2007). Personality traits, which are shaped by values and beliefs, are crucial in guiding entrepreneurial decision-making. As a result, investigating this underlying relationship by combining various concepts will provide an insight into the relationships. Thus, the first research question of the study is what is the impact of big five personality traits (BFPT) on entrepreneurship intention (EI)? The first objective of the study is to investigate BFPT and EI.

The importance of having an entrepreneurial mindset (EM) has gained a lot of attention (Brown et al., 2011; Cui, 2021; Pidduck et al., 2021). Entrepreneurial mindset is conceptualized as the inclination for entrepreneurship is based on the way of being critical and abilities of being critical thinkers (Nabi et al., 2017; Liao et al., 2022). Individuals that have a more EM are more likely to seek out and exploit new chances and innovations (Burnette et al., 2019). Nevertheless, impact of EM on entrepreneurial intention (EI), on the other hand, has to be confirmed further (Hallak et al., 2011; Chien-Chi et al., 2020; Handayati et al., 2020; Cui, 2021; Liao et al., 2022). Previous research has shown that having an EM can help in developing more dynamic skills and competencies. Very few studies have examined the role EM on EI. Therefore, the second objective of this study is to investigate the impact of EM on EI.

A variety of factors influence one’s decision to pursue entrepreneurship as a career including self-efficacy (Krueger and Carsrud, 1993), social context (Henley et al., 2017; Bellò et al., 2018), education (Shahab et al., 2018). In addition, self-efficacy (SE) decides whether or not you want to be an entrepreneur (Ryan, 1970). Moreover, SE is the determination required to generate a result that is closest to action or action intentionality Bandura (1986). Self-employment highly depends on these perceptions of self-efficacy (Scherer et al., 1989), which can be applied to predict the EI. In addition to that, SE has been identified as a critical antecedent in the creation of EIs in several research (Wilson et al., 2007). SE was found to be a significant predictor of EIs and/or activity (Fitzsimmons and Douglas, 2011). Within the context of entrepreneurship, the role of personality traits in deciding on a career path is also studied within the attraction-selection-attrition (ASA) framework as this framework posits that people like to work in situations with others who have similar personality characteristics to them (Schneider et al., 2000). This study attempts to fill the multiple gaps. First, the BFPT, as well as entrepreneurial self-efficacy (ESE), are considered determinant elements in an individual’s EI in this study which are rarely studied together in literature and most of the studies have advocated to investigate the relationship among them (Caprara et al., 2010; Şahin et al., 2019; Elnadi and Gheith, 2021). This study focuses on these personal characteristics based on a large body of evidence that the BFPT and ESE play a predictive role in EI (Zhao and Seibert, 2006). Previous empirical studies produced mixed outcomes on the study of individual personalities and their EI (Baron et al., 2001; Zhao and Seibert, 2006; Şahin et al., 2019; Cui, 2021).

Psychological characteristics are linked to business formation and success, according to meta-analytic evaluations (Frese and Gielnik, 2014). Such traits impact individual’s willingness to engage in entrepreneurial activity. Yet, several entrepreneurship studies initially concluded that psychological personality assessments were ineffective (Brandstätter, 2011). With the emergence of meta-analytic studies in entrepreneurship and big-five personality traits linked to entrepreneurial goals, this assumption changed (Zhao and Seibert, 2006; Cui, 2021). According to psychologists, attitude, which serves as the foundation for a person’s opinion and justification of conduct, has a significant impact on individual intentions (Ferreira et al., 2012). As a result, it’s necessary to look into this link (Fai et al., 2017; Cui, 2021). After reviewing the literature, it has been found that attitude has a significant role in university students’ desire to start a business or enterprise (Urbano et al., 2017; Bazkiaei et al., 2020). Personality traits have been widely addressed among individual variations; however, only a few empirical research have looked at how these traits effects on EI among students (Hu, 2008). Education industry plays a significant role in the EI (Bazkiaei et al., 2020). To fill this second research gap, this research study intends to investigate the role of BFPT and EM on EI among students. Another gap this study intends to fill is to investigate the role of EM on attitude toward entrepreneurship (ATE). Similarly, entrepreneurial passion (EP) is very crucial in the EI (Cui, 2021). Moreover, according to (Bierly et al., 2000), the EP is a strong emotion that has the potential to help people reach their full potential. Moreover, enthusiasm drives people to pursue their dreams of starting their own business which is entrepreneurial activities. Very limited number of studies check the EP as a moderator. Third, this study fills the gap by investigating the moderating role of the EP on the relationship between ATE, ESE, and EI. Moreover, studies have highlighted to investigate the above relationships in Saudi Arabia context (Naushad, 2018; Ali et al., 2019; Al-Mamary et al., 2020; Elnadi and Gheith, 2021). Therefore, fourth, this study fills the gap by extending the existing literature of BFPT and entrepreneurial mindset by providing the empirical evidence from developing country context (Saudi Arabia), and data were collected from students from the Kingdom of Saudi Arabia (KSA) including both private and public universities.

**Figure 1:**
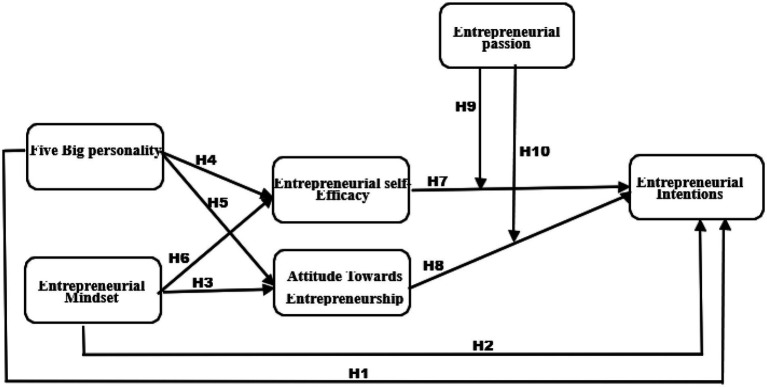
Conceptual framework of the study.

The framework of this research study has been developed after a through literature review, in this framework, the impact of BFPT has been examined on EI directly and EM on EI directly and indirectly through the ESE and ATE. More interestingly, the moderating role of EP has also been investigated which were highlighted by multiple research studies. This framework in Figure 1, is unique of its kind that it has been developed by combining the holistic research studies and based on multiple research gaps which are discussed in the above paragraphs. In total 10 hypotheses are developed to test the framework of the study.

## Literature review

### Theory of planned behavior

The elements of this theory of planned behavior (TPB), the first is perceived desirability, which refers to a person’s attitude toward entrepreneurship (ATE) or level of interest in it. When faced with a number of problems and options, a person can become overwhelmed and may decide whether or not to react based on an early assessment of the conduct (Ajzen, 1991).

### The big five personality traits and entrepreneurial intention

In order to describe major personality traits, a comprehensive model known as the big five models was constructed as human personality is complex broad categories (Goldberg, 1990). The five factors received widespread support after the model was introduced-conscientiousness, openness to experience, emotional stability, extraversion, and agreeableness – proposed by the model causing the big five to be the most often used personality locus (Brandstätter, 2011).

A lot of research studies have focused on whether the BFPT have an impact on EI (Zhao and Seibert, 2006; Bazkiaei et al., 2020; Awwad and Al-Aseer, 2021; Biswas and Verma, 2021; Huang et al., 2021; Xie et al., 2021). A brief review of the literature is provided below to support the relevance of the BFPT to EI.

Conscientiousness. It can be referred to Individuals with usually having the qualities of working hard, planning well, remaining organized and ready when asked to perform duties and tasks (Costa Jr and McCrae, 1992; Zhao and Seibert, 2006; Ariani, 2013; Hossain et al., 2021). Entrepreneurship and conscientiousness are closely related. A person who has a strong desire to be successful and remained motivated toward achieving their set goals tend to have more traits of an entrepreneur (McClelland, 1961; Baum and Locke, 2004). Entrepreneurs are people who dislike doing the same thing over and over again, who take personal responsibility for their actions and desire to see tangible outcomes of their choices, actions, and decisions (Antoncic et al., 2015). In personality studies, conscientiousness was found to be a trait which actually differentiates managers from entrepreneurs (Zhao and Seibert, 2006). The meta-analysis study conducted by Zhao et al. (2010) highlighted that conscientiousness was found to be consistent and vital dimension that is closely related to EI. In addition to that, another study found no significant differences in conscientiousness between those who take the initiatives as entrepreneurs and those who do not take such type of initiatives which are non-entrepreneurs (Antoncic et al., 2015; Wang et al., 2016).

Openness to experience. This big five model dimension is defined as well as a person’s inquisitiveness for taking initiative with new ideas, concepts, and the value system as well as their desire to strive for novel, unusual, and unique (Zhao and Seibert, 2006; Ariani, 2013; Cui, 2021). Those individuals with a high amount of openness to experience score are likely to be more in terms of creativeness and imagination while thinking in a different way to try novel things (Liang et al., 2013; Cui, 2021). An entrepreneur is a person who is efficient and innovative, according to Schumpeter (1934). Openness to new experiences shows the clear distinction between the professionals and entrepreneurs as it is based on emotional stability and extraversion which is referred as vital element (Chen et al., 2015). Openness to new experiences was the second most strongly linked personality trait to the desire to start a business (Zhao et al., 2010).

Emotional stability. When individuals are in state of relax and remained clam during the tough time or the inconvenient times, they are considered emotionally stable. Emotions that bring the negative energy in form of anxiety, fear of loss, or fear of unknown lead to the emotional instability (Costa and McCrae, 1992). Scholars and practitioners highlighted that in order to start the new venture or any type of the business, the confidence level, ability to handle the pressure, and resilience to perform different activities in difficult times are based on emotional stability (Baron and Markman, 1999; Zhao and Seibert, 2006; Al-Hammadi and Moore, 2021). Regarding this particular trait, there is a variety of results. A study highlighted that they did not find a significant difference in neuroticism between entrepreneurs and nonentrepreneurs, according to the study conducted by Antoncic et al. (2015).

Extraversion. Individuals with high level of extraversion tend to be more pleasant, friendly, gregarious, lively, moreover, they have the tendency to be dominating and assertive in social circle. Assertion means claim and persuasion in terms of influence are typically displayed by those with high-level communication capabilities and social impact (Baum et al., 2014; Awwad and Al-Aseer, 2021). Entrepreneurs need to organize and manage their subordinates and teams, in order to encourage their innovative business concepts to employees and customers (Shane, 2003) and extraverts are more likely to find this easier than introverts. Despite this, earlier research on the trait of extraversion in entrepreneurs has been inconclusive (Zhao and Seibert, 2006; Zhao et al., 2010). In addition to that, in a meta-analysis, no significant difference was found between managers and entrepreneurs (Zhao and Seibert, 2006).

Agreeableness. Individuals with higher level of agreeableness tend to be having the attributes of trusting, altruistic, compassionate, and quality of forgiveness (Zhao and Seibert, 2006). In addition to that, entrepreneurs are considered to be more cooperative and supportive yet for such attributes, the level of high motivation and energy is required (Antoncic et al., 2015; Laouiti et al., 2022). Another study highlighted that one of the main attributes of the entrepreneur is to develop trust building measure with team members as well as with all stakeholders (Eisenhardt and Schoonhoven, 1990; Shane and Cable, 2002), further, they highlighted that entrepreneur also must build trust with their customers. According to empirical research, being agreeable is related with a lower likelihood of becoming an entrepreneur (Wooten et al., 1999). Despite the findings of Zhao et al. (2010) in meta-analysis, there was no evidence of a link between the Big Five model’s agreeableness construct and entrepreneurial intent. As a result, we suggest the following hypothesis.

*H1*: BFPT is positively associated with EI.

### Entrepreneurial mindset and entrepreneurial intention

One of the most significant predictors of entrepreneurial behavior has been identified as EI resulting in the establishment of new enterprises (Liñán, 2004; Souitaris et al., 2007; Prodan and Drnovsek, 2010; Bögenhold and Fachinger, 2010, 2014; Dieter and Uwe, 2011; Jiatong et al., 2021; Mukhtar et al., 2021). Entrepreneurial intention, according to DeNoble et al. (1999a), is the entrepreneur’s natural knowledge, propensity, and behavioral proclivity to start a new business. Study by Thompson (2009), EI is the belief that entrepreneurs want to start a firm. To put it another way, entrepreneurs are the individuals whose intentions are primarily focused with entrepreneurial outcomes which are only business centric (Darmanto and Yuliari, 2018; Kong et al., 2020). Other researchers claim that mindset and mentality are a broader vision that is used to make new recommendations, assess risks and opportunities related to have new business initiatives depends on the border perspective of individual perception rather than in a particular way or features (Haynie et al., 2010; Davis et al., 2016; Roeslie and Arianto, 2022). Previous research has found that the association between entrepreneurial attitude and ambition to be an entrepreneur has a beneficial effect (Burke and Aldrich, 1984; Bowen and Hisrich, 1986; Hisrich, 1992; Kim and Aldrich, 2005; Aldrich, 2012; Walter and Block, 2016; Hisrich and Ramadani, 2017; Cui and Bell, 2022). As a result, this study suggests the following hypothesis.

*H2*: Entrepreneurial mindset is positively associated with EI.

### Big five personality traits and entrepreneurial self-efficacy

It also necessitates success in responsibilities such as invention, marketing, management, and finance that are associated with the start-up of a new enterprise (Chen et al., 1998; Hsu et al., 2017; Şahin et al., 2019; Chien-Chi et al., 2020). Individuals’ personality traits have a significant impact on their self-efficacy (Stajkovic et al., 2018). The BFPT have been linked to SE in various studies (Judge et al., 2007; Cristina et al., 2018; Coco et al., 2019; Hua et al., 2020; Cui, 2021), Extraversion, openness, agreeableness, and responsibility are positively associated with SE, while neuroticism is adversely correlated (Judge et al., 2007). Some researchers found that individuals who scored better on conscientiousness had stronger self-efficacy views (Brown et al., 2011; Chien-Chi et al., 2020). Openness transforms requests into challenges to be met, resulting in increased level of engagement in different tasks and their self-efficacy (Sanchez-Cardona et al., 2012; Neneh, 2020). According to research, agreeableness might lead to higher self-efficacy (Coco et al., 2020). Individual SE is positively connected with extraversion and adversely correlated with neuroticism, according to some studies (Schmitt, 2007). Djigić et al. (2014) revealed that conscientiousness can be referred as a vital predictor of teacher’s SE, although another study by Marcionetti and Rossier (2016) highlighted that the association between the conscientiousness, extraversion, and neuroticism is closely related and positive. Additionally, other researchers advocated that conscientiousness and extraversion lower down the neuroticism and enhance the self-efficacy (Brown and Cinamon, 2016). As a result, this study suggests the following hypothesis.

*H4*: BFPT is positively related to ESE.

### Big-five personality trait and attitude toward entrepreneurship

Individual persistent aims toward entrepreneurship are referred to as attitudes; which could be either have a positive or negative status and be influenced by the environment. According to Hu (2008), there is a positive association between (Agreeableness, extraversion, conscientiousness, and openness to new experiences are some of the Big-Five personality traits.) Neuroticism has a detrimental impact on entrepreneurial attitude (based on experience). Previous research has found to be one of major predictor of EI is one’s ATE (Costa Jr and McCrae, 1992; Autio et al., 2001; Duong, 2021; Huang et al., 2021, 2022; Srivastava et al., 2021). Personality traits can have an impact (Luthje and Franke, 2003; Hamza et al., 2021). As a result, this study suggests the following hypothesis.

*H5*: BFPT is positively associated with ATE.

### Entrepreneurial mindset and antecedents

It can be referred as taking unusual decisions in uncertain circumstances which require different and unique kind of thinking and judgments (Solesvik et al., 2013). Moreover, EM, according to Zupan et al. (2018), highlighted that it involves not only the related experience, creativity in solving a problem, identifying the new ways of doing it along with identification of opportunity, but it also contains the way the entrepreneur thinks or thinking. Psychology, particularly personality psychology, is intrinsically tied to the EM (Solesvik et al., 2013). The creation of an EM was discussed by Westhead and Solesvik (2016), who affirmed that it is related with the ability to think creatively, to look for possibilities rather than problems, and to provide solutions rather than complain (Naumann, 2017; Cui, 2021; Daspit et al., 2021; Kuratko et al., 2021). Examining an entrepreneur’s level of ESE is one way for them to better understand their own motivations, capabilities, and limitations, because ESE allows them to assess their own competency in carrying out entrepreneurial activities (McGrath and MacMillan, 2000; Ngek, 2015; McMullen and Kier, 2016; Karyaningsih et al., 2020; Cui, 2021). As a result, based on the information provided, the hypotheses are:

*H3*: EM is positively associated to ATE.

*H6*: EM is positively associated to ESE.

### The role of attitude toward entrepreneurship on entrepreneurial intention

The level of being attracted toward entrepreneurship behavior and the belief system that allows one to take certain actions that will result in a positive outcome is referred to as one’s ATE. This was defined by Liñán and Chen (2009) as preferences and benefits or downsides, respectively. Whereas others described It’s a mindset for becoming an entrepreneur (Maes et al., 2014). One’s attitude toward entrepreneurial behavior, according to Lee-Ross (2017), is a general assessment of that behavior, or whether it is favorable or not. Previous studies have revealed a statistically significant link between EI and ATE. Demonstrating that students see entrepreneurship as an enticing, desirable career option, and that if given the opportunity and resources, they would pursue entrepreneurial companies (Ajzen, 2001, 2005, 2011; Rauch and Frese, 2007; Ajzen and Cote, 2008; McGee and Peterson, 2017; Shah et al., 2020; Huang et al., 2021, 2022; Palmer et al., 2021; Yasir et al., 2021; Yousaf et al., 2021). Based on previous studies, this study proposes the following hypothesis:

*H8*: ATE is positively associated to EI.

### The role of self-efficacy on entrepreneurial intention

ESE has been described in a variety of ways by researchers. The idea of “self-efficacy” was defined by Bandura (1977) as an individual’s belief in their talents Matsu aptitudes to execute specific tasks or assignments. The actions which are based on self-motivation, environment, and perception are depicted in this idea. It is a person’s belief in their potential to start a successful business enterprise (McGee et al., 2009). ESE, according to Dissanayake (2013), is an individual’s ability or talent to improve motivation, cognitive resources, and particular set of action plan that are essential in order to be successful in particular profession. As a result, ESE is an important cognitive predictor of entrepreneurial purpose and activity (Laviolette et al., 2012). Previous research has demonstrated that it helps people become entrepreneurs (Bandura, 1982; Oyugi, 2015; Utami, 2017; Elnadi and Gheith, 2021; Pelegrini and de Moraes, 2021; Yousaf et al., 2021). Based on above information, this study presents the following hypothesis:

*H7*: ESE is positively associated to EI.

### The potential moderator effect of entrepreneurial passion

EP is the inspiration that drives people to pursue entrepreneurial endeavors (Bierly et al., 2000; Cardon and Stevens, 2009). It’s also a powerfully good emotion that has the potential to help people reach their full capacity (Baron and Ward, 2004; Cardon and Stevens, 2009). EP instills the courage to take risks and overcome challenges as a result of a love for business that expresses both emotionally and cognitively (Baron and Ward, 2004; Cardon and Stevens, 2009). EP has the power to influence entrepreneurship thoughts, i.e., it has a significant impact on ESE (Schwarz et al., 2009; Cardon and Kirk, 2013; Donaldson and Campbell, 2019; Feng and Chen, 2020; Anjum et al., 2021; Lee and Herrmann, 2021; Montiel-Campos, 2021; Newman et al., 2021). Furthermore, enthusiasm has been shown to enhance confidence and competence in the context of particular activities and aims (Cardon et al., 2013; Anjum et al., 2021; Bignetti et al., 2021). Few research studies have looked at the role of entrepreneurial enthusiasm in moderating cognitive antecedents. In undergraduate level students’ persistent EI, entrepreneurial enthusiasm showed a significant positive moderating influence on AT, perceived appeal, and perceived feasibility (Tehseen and Haider, 2021). Individuals with EP may have a good perception of the outcomes of entrepreneurship. Hence, the following hypotheses are formed:

*H9*: EP moderates the relationship between ESE and EI.

*H10*: EP moderates the relationship between ATE and EI.

## Research methodology

In this study, the hypothesized relationships were checked, the type of the study is quantitative through deductive approach while laid the foundation on the philosophical perspective of positivism. Additionally, respondents were students from the Kingdom of Saudi Arabia (KSA) including both private and public universities. The authors drew students from a wide range of academic disciplines, including business, economics, accounting, MIS, finance, and computer science. The authors contacted them *via* an online questionnaire with the help of faculty members. A survey link long with informed consent was provided *via* email to 390 students. The authors stated unequivocally that all information provided by our responders would not be disclosed. The samples were randomly selected. Emails of reminder were sent to all respondents who did not respond within due time of 3 weeks of receiving the survey link received. Finally, a total of 270 respondents responded to the questionnaire. Resulting in a response rate of 69%. Males made up the majority of the responders 65.63% and females 35.37%. Furthermore, 45.17% of the respondents had only temporary employment experience, while 54.83% were students.

### Instruments and measures

For data collection, the survey approach was sued and it was the primary source of information. This study adapted the scales which are already existing to measure the concepts because prior research had demonstrated them to be valid and reliable. The BFPT were measured in this study using (TIPI) by Gosling et al. (2003), agreeableness, conscientiousness, extraversion, openness to experience, and emotional stability. To measure EM, the six-item scale is used by researchers which was introduced by Handayati et al. (2020). To assess the ESE, researchers used scale which were introduced by DeNoble et al. (1999b), and Liñán (2008). Moreover, four-item scale was used and adapoted from Liñán et al. (2011) to measure ATE. While to measure EP, researchers used five-items introduced by Biraglia and Kadile (2016). Lastly, to assess the EI, researchers used the scale introduced by Liñán et al. (2011).

## Data analysis

### Students’ demographic characteristics

According to the data gathered on gender-based received from students, males made up more than three-quarters of the total population of respondents (i.e., 65.63%), and females made up the rest of the group (i.e., 35.37%). As of age wise of students grouping, the majority of them are young age. The age group of 20–29 years accounted for 56.08 percent of all students. While the next largest category was found to be students above the age of 18. Only 17.03 percent and 4.97 percent of those aged 30–39 and 40–49 years, respectively, were found. When it comes to the students’ educational qualifications, Three-quarters of the population was expected to have a bachelor’s degree. Furthermore, more than 63 percent of all respondents took the subjects which are related to management and business as per their regular teaching course load according to their higher education requirements at various Saudi Arabian universities.

### Assessment of measurement model

Convergent and discriminant validity were investigated using a series of confirmatory factor analyses (CFAs; Hair et al., 2019). While estimating the measurement model the four procedures be followed namely, internal consistency, composite reliability, indicator reliability, convergent, and discriminant validity. For internal consistency, composite reliability values were larger than 0.80, exceeding the minimum criteria of 0.70, and indicating internal consistency (Hair et al., 2014). All of the items had loadings over the cutoff value and they were all retained. The average variance extracted (AVE) of each component was examined using a threshold value of 0.50 to determine convergent validity (Hair et al., 2019). In this study, results supported the convergent validity as range is within the threshold as shown in Tables 1–3.

**Table 1:** Descriptive statistic.

Variables	Mean	Maximum	Minimum	Number	SD
BFPT	3.50	5	1	270	0.956
EM	3.66	5	1	270	0.774
ESE	3.48	5	1	270	0.921
ATE	3.64	5	1	270	0.870
EP	3.60	5	1	270	0.758
EI	3.07	5	1	270	0.664

ESE, entrepreneurial self-efficacy; BFPT, Big-Five Personality Trait; EI, entrepreneurial intention EE; EM, entrepreneurial mindset; ATE, attitude toward entrepreneurship.

**Table 2:** Evaluation of the measurement model.

Construct items	Number of dimensions	Factor loading	AVE	CR	Cronbach’s alpha
Big-Five Personality Trait (BFPT)	BFPT 1	0.71	0.712	0.831	0.85
	BFPT 2	0.72			
	BFPT 3	0.81			
	BFPT 4	0.72			
	BFPT 5	0.85			
	BFPT 6	0.63			
	BFPT 7	0.75			
	BFPT 8	0.80			
	BFPT 9	0.79			
	BFPT 10	0.83			
Entrepreneurial mindset (EM)	EM 1	0.81	0.709	0.857	0.82
	EM 2	0.83			
	EM 3	0.74			
	EM 4	0.78			
	EM 5	0.74			
	EM 6	0.71			
Entrepreneurial self-efficacy (ESE)	ESE 1	0.87	0.755	0.841	0.81
	ESE 2	0.82			
	ESE 3	0.72			
	ESE 4	0.61			
	ESE 5	0.69			
	ESE 6	0.77			
Attitude toward entrepreneurship (ATE)	ATE 1	0.71	0.682	0.880	0.88
	ATE 2	0.79			
	ATE 3	0.68			
	ATE 4	0.81			
Entrepreneurial passion (EP)	EP 1	0.80	0.731	0.812	0.89
	EP 2	0.79			
	EP 3	0.73			
	EP 4	0.75			
	EP 5	0.82			
Entrepreneurial intention (EI)	EI 1	0.88	0.742	0.815	0.82
	EI 2	0.85			
	EI 3	0.75			
	EI 4	0.71			
	EI 5	0.73			
	EI 6	0.69			

AVE, average variance extracted; CR, composite reliability.

**Table 3:** Discriminant validity.

Constructs	ESE	BFPT	EI	EM	ATE
ESE	**0.859**				
BFPT	0.236	**0.713**			
EI	0.314	0.314	**0845**		
EM	0.316	0.214	0.412	**0.784**	
ATE	0.277	0.218	0.531	0.32.3	**0.824**

Diagonal elements (in bold) are the square root of AVE. Elements below the diagonal are the correlations among constructs. ESE, entrepreneurial self-efficacy; BFPT, Big-Five Personality Trait; EI, entrepreneurial intention EE; EM, entrepreneurial mindset; ATE, attitude toward entrepreneurship.

The Fornell-Larcker criterion (Fornell and Larcker, 1981) and the Heterotrait-Monotrait (HTMT) ratio were used to assess discriminant validity (Hair et al., 2019). Moreover, all of the AVEs on the diagonals in Table 3 were bigger than the corresponding row and column values, showing that the measures were discriminant. All HTMT ratio values in this investigation were less than the crucial value of 0.85, as determined by the cut-off value of 0.85 for proving discriminant validity. Results confirm the measurement model.

### Multicollinearity and common method bias

This work used the software PLS to perform a full collinearity test (Kock and Lynn, 2012), to analyze collinearity simultaneously (Kock and Gaskins, 2014; Xie et al., 2021). In Table 4, all of the values of VIF are less than 3.3, and all values of tolerances are greater than 0.10 which means they are in acceptable range. The whole collinearity test process appears to be successful in identifying common method bias (CMV). In addition to that, currently, the most used technique for examining CMV is the Harman single-factor test. According to our research, the characteristic root of the common factor with the highest explanatory power in the absence of factor rotation is 10.256, which accounts for 40.145 percent of the total variance. The majority of the covariance between independent variables and dependent variables cannot be explained by a single factor. It demonstrates that this study is free from significant CMV (Podsakoff et al., 2003; Huang et al., 2021, 2022; Xie et al., 2021).

**Table 4:** Multicollinearity.

Constructs	Tolerance	VIF
Big-Five Personality Trait (BFPT)	0.714	1,554
Entrepreneurial mindset (EM)	0.837	2,354
Entrepreneurial self-efficacy (ESE)	0.771	1,358
Attitude toward entrepreneurship (ATE)	0.925	1,256
Entrepreneurial intention (EI)	0.654	1,365
Entrepreneurial passion (EP)	0.721	2,258

VIF, variance inflation factor.

### Hypotheses testing

The hypotheses were tested using PLS-SEM. The structural model was examined using the coefficient of determination (*R*^2^), path coefficient (*β*), values of *p*, and effect sizes (*f*^2^) with a bootstrapping approach involving 5,000 sub-samples suggested by Hair et al. (2019). In addition, in response to recent critiques that simply using values of *p* to test hypotheses is insufficient, this study used values of *p* with confidence ranges and effect sizes as additional criteria (Hahn and Ang, 2017). As a result, reliable and adequate criteria were developed to assess the hypotheses, as illustrated in Table 5.

**Table 5:** PLS hypothesis testing.

Hypotheses	*β*	SE	*t*-value	*p*-value	LLCI	ULCI	Result
H1: Big-Five Personality Trait → Entrepreneurial intention	*0.367*	0.072	3.254	0.000*	0.062	0.335	Supported
H2: Entrepreneurial mindset → Entrepreneurial intention	*0.333*	0.061	2.587	0.002*	0.054	0.451	Supported
H3: Entrepreneurial mindset → Attitude toward entrepreneurship	*0.252*	0.062	3.562	0.000**	0.061	0.363	Supported
H4: Big-Five Personality Trait → Entrepreneurial self-efficacy	*0.258*	0.059	4.258	0.003*	0.163	0.314	Supported
H5: Big-Five Personality Trait → Attitude toward entrepreneurship	*0.262*	0.069	3.897	0.000**	0.184	0.391	Supported
H6: Entrepreneurial mindset → Entrepreneurial self-efficacy	*0.271*	0.045	2.985	0.002*	0.091	0.299	Supported
H7: Entrepreneurial self-efficacy → Entrepreneurial intention	*0.472*	0.661	3.324	0.000**	0.224	0.399	Supported
H8: Attitude toward entrepreneurship → Entrepreneurial intention	*0.302*	0.055	4.562	0.002*	0.642	0.301	Supported

ESE, entrepreneurial self-efficacy; BFPT, Big-Five Personality Trait; EI, entrepreneurial intention EE; EM, entrepreneurial mindset; ATE, attitude toward entrepreneurship.

*p ≤ 0.05, **p ≤ 0.01.

The value of *R*^2^ for two endogenous latent constructs are 0.358 for the ESE and 0.337 for ATE which are in the range of acceptable and are moderate values (Hair et al., 2016). Furthermore, this study used a blindfolding process to test the predictive relevance. As shown in Table 5, all of the effects are positive and significant at the 1% level or higher. The values of *f*^2^ can be small, medium, or large with cutoff value range is 0.02, 0.15, or 0.35 (Hair et al., 2012, 2016). In terms of relationship of BFPT on EI, the H1 states, there is a positive relationship of FBPT on EI and was found to have positive and significant effect on EI (*β* = *0.367, f*^2^ = 0.041, *p* < 0.001), thus H1 is supported, while the H2 states that there is a positive relationship of EM on EI and was found to have positive and significant effect on EI (*β = 0.333, f*^2^ = 0.032, *p* < 0.001). Therefore, H2 is supported.

In terms of the influence of BFPT on ESE and ATE, the H4 states that BFPT is positively related to ESE and was found to have a significant effect on ESE (*β* = *0.258, f*^2^ = 0.031, *p* < 0.001). Thus, H4 is supported. Moreover, H5 states that BFPT has a significant positive impact on ATE (*β* = *0.262, f*^2^ = 0.051, *p* < 0.001). Therefore, H5 is also supported.

The effect of the EM on ESE which is H6 and the effect of the EM on ATE which is H3, both state that EM is positively related to ESE and was found to have a positive effect on ESE (*β* = *0.271*, *f*^2^ = 0.0612, *p* < 0.001), thus H6 is supported. Similarly, the H3 states that EP is positively related to ATE and was also found to have a positive effect on ATE (*β* = *0.252, f*^2^ = 0.055, *p* < 0.001), Thus, it is supported.

While the relationship between the ESE and ATE on EI, Table 6 shows that ESE has strong and positive effect on EI (*β* = *0.472, f*^2^ = 0.151, *p* < 0.001), thus H7 is supported. Furthermore, Table 5 shows that ATE was found to have positively related to EI (*β* = *0.302, f*^2^ = 0.154, *p* < 0.001). Thus, supported H8.

**Table 6:** Moderation tests (indirect effects).

Hypotheses	*β*	SE	*t*-value	*p*-value	LLCI	ULCI	Result
H9: Entrepreneurial passion * Entrepreneurial self-efficacy → Entrepreneurial intention	0.152	0.059	3.587	0.000***	0.051	0.225	Supported
H10: Entrepreneurial passion * Attitude toward Entrepreneurship → Entrepreneurial intention	0.132	0.066	2.547	0.000***	0.060	0.192	Supported

ESE, entrepreneurial self-efficacy; BFPT, Big-Five Personality Trait; EI, entrepreneurial intention EE; EM, entrepreneurial mindset; ATE, attitude toward entrepreneurship.

***p ≤ 0.001; ns, not significant.

Moderation analyses were carried out by using the SPSS PROCESS macro (Hayes, 2013), presented in Table 6. Hypothesis H9 hypothesized that EP positively moderators the relationship between ESE and EI, which is also supported (*β* = 0.152, *t* = 3.587, 95% bias-corrected CI = [0.051, 0.225]). Similarly, the H10 hypothesized that EP positively moderators the relationship between ATE and EI, which is also supported (*β* = 0.132, *t* = 2.547, 95% bias-corrected CI = [0.060, 0.192]).

## Discussion and conclusion

First, this study further provides the evidence of significance of FBPT and EM impact on students’ ESE, and ATE, all of which help to support EIs. According to the findings of this study, FBPT and EM positively influence the EIs which is consistent with the previous studies (Wang et al., 2016; Tehseen and Haider, 2021).

Second, according to the findings of this study, FBPT and EM positively influence the ATE (Ayuni, 2018; Wardana et al., 2020; Cui, 2021; Pidduck et al., 2021; Liao et al., 2022). This finding highlights the importance of FBPT and EM in influencing students’ entrepreneurial goals by altering their attitudes toward entrepreneurship. Additionally, entrepreneurial attitudes influenced desires to become entrepreneurs significantly more in students who have FBPT and EM than the students who did not have the FBPT and EM. Similarly, FBPT and EM were found to have positive effect on ESE. As compared to the previous studies, this study offers, to help students develop a better grasp of entrepreneurial activities, this research suggests that FBPT and EM programs should be included as part of their projects and classes in order to build and cultivate the FBPT and EM. It is recommended to provide guidance on how to use a variety of specific skills and tactics to increase student self-efficacy when engaging in entrepreneurial activity.

Third, our findings revealed a significant positive link between entrepreneurship attitude and EI. Our findings are consistent with previous research studies and advocate that ATE is the strongest predictor of EI (Liñán and Chen, 2009). According to the findings, students would consider entrepreneurship to be a desirable and advantageous career option, and would pursue entrepreneurial ventures. As compared to the previous studies, this research reveals that ESE has a significant impact on EI consistent with previous findings (Baidi and Suyatno, 2018; Yamina and Mohammed, 2019; Hassan et al., 2020). As a result, when students have more belief in the success of entrepreneurship, they are more likely to contribute to entrepreneurial initiatives.

Fourth, another interesting finding from the study is the EP has a partial moderating influence on the relationship between ESE and ATE and EI. This finding is consistent with the studies conducted by Liao et al. (2022) and Tehseen and Haider (2021). It supports the notion that Entrepreneurship is a module should be placed in a dual/triple degree program for students. They develop positive entrepreneurial attitudes, consider themselves to be more appealing and capable of commencing a long-term entrepreneurial venture and their enthusiasm improves their EM and feasibility of starting a long-term enterprise.

This study provided a full research framework to assess three research questions that had yet to be addressed by previous research. With several contributions, the importance of BFPT and mindset in generating EI among university students is highlighted in this study. The EI is accelerated by EP as a moderator, according to the findings of this study. These findings could be used by decision-makers as a point of reference.

## Implications of the study

This study’s findings have a number of academic and management implications. In academic context, when new antecedents are given to define an individual’s conduct, TPB can be utilized as a model to examine diverse profiles of entrepreneurial behavior and as a solid foundation to investigate its moderating influence. The findings of our research add to the theoretical perspectives of Gorman et al. (1997) and Kuratko (2005), proving that entrepreneurship education may lead to students pursuing their entrepreneurial career goal which could lead to successful start-ups after completing the graduation studies (Peterman and Kennedy, 2003; Zhang et al., 2014). In addition to that, the empirical evidence of this study support the assumption that an individual’s self-efficacy, along with his or her skill for pursuing motivation, and a plan of action, will be a crucial factor in the formulation of entrepreneurial goals, in accordance with SCCT.

In terms of the practical implications, BFPT and EM can enhance the EI in multiple ways. Two ways that FBPT and EM influence students’ EI are ESE and ATE (Arshad et al., 2016; Buana et al., 2017; Shah et al., 2020; Liao et al., 2022). Our findings support the idea that self-efficacy plays a significant role in the development of EI. As SCCT evolves, ESE is becoming more important in establishing entrepreneurial intent. Similarly, our research highlights the direct favorable impact of entrepreneurship attitudes on EI. As a result of our findings, the TPB (Ajzen, 1991) appears to be a viable theoretical framework for analyzing an individual’s EI.

### Limitations and future directions

There are various limitations to this study that indicates areas where future research should be pursued. First, conducting the study with a pre- and post-test approach would have been fascinating (Rideout and Gray, 2013), such that differences in EI can be investigated different BFPT and entrepreneurial mind. Another limitation of this study is that it is cross-sectional in nature, longitudinal research should be carried out in future studies to investigate the changes in entrepreneurial attitudes and ambitions over time, as well as the development of new businesses of entrepreneurial conduct that is motivated by a desire to succeed. Third, to investigate future studies should investigate the EI with other moderator and mediator such as entrepreneurial leadership and entrepreneurial training, and entrepreneurial practice.

## Data availability statement

The original contributions presented in the study are included in the article/supplementary material, further inquiries can be directed to the corresponding author.

## Author contributions

SS and BA-G worked on the overall paper (introduction and literature review and developing the conceptual framework). MS worked on the paper methodology and data analysis. All authors contributed to the article and approved the submitted version.

## Conflict of interest

The authors declare that the research was conducted in the absence of any commercial or financial relationships that could be construed as a potential conflict of interest.

## Publisher’s note

All claims expressed in this article are solely those of the authors and do not necessarily represent those of their affiliated organizations, or those of the publisher, the editors and the reviewers. Any product that may be evaluated in this article, or claim that may be made by its manufacturer, is not guaranteed or endorsed by the publisher.
